# Nutrient digestibility and nitrogen balance in different pig breeds fed raw, sprouted, or roasted (*Vigna unguiculata*) diets

**DOI:** 10.1007/s11250-023-03742-w

**Published:** 2023-09-28

**Authors:** M. W. Lubisi, J. J. Baloyi, F. Fushai

**Affiliations:** Department of Animal Science, School of Agriculture, University of Venda, Private Bag X5050, Thohoyandou, 0950 Limpopo South Africa

**Keywords:** Cowpea processing, Windsnyer, Large White, Landrace, Nitrogen utilization

## Abstract

The study examined effects of feeding iso-nutrient (150 g CP, 17.3 MJ ME kg^−1^) raw (RCP), sprouted (SPC), or roasted (RSCP) cowpea diets to Windsnyer (W), Large White (LW) × Landrace (LR), and the 3-way crossbred (W × LW × LR) growing pigs. Diet dry matter (DM) digestibility was estimated using standard, 3-step (gastric, small intestines, colon) in vitro digestion. Dietary in vivo nutrient digestibility and nitrogen balance were evaluated using 3 weaned pigs of each genotype. Pigs were housed in individual metabolic cages. The diets were assigned to pigs in a 3 × 3 change-over factorial experiment within three balanced, 3 × 3 Latin squares. Feeding periods consisted of 7 days adaption + 5 days’ measurement of feed intake, and the total faecal and urine excretions. The SPC diet increased step 3 in vitro DM digestibility compared to RSCP (*P* < 0.05). Metabolic size-scaled feed consumption was higher on the RCP compared to the RSCP diet (*P* < 0.05). Cowpea processing reduced apparent DM and ash digestibility (*P* < 0.05). LW × LR pigs exhibited lower digestibility of ash and acid detergent fiber (ADF) compared to 3-way crossbred pigs (*P* < 0.05). Significant genotype-diet interactions were observed for nitrogen intake (*P* < 0.0001), digestible nitrogen (*P* = 0.043), urinary nitrogen output (*P* < 0.0001), faecal nitrogen output (*P* < 0.0001), total nitrogen excretion (*P* < 0.0001), and nitrogen retention (*P* < 0.001). The biological value of feed protein was higher for W pigs than 3-way crossbred pigs (*P* < 0.05). Genotype-diet interactions suggested unique digestive and, or metabolic adaptive traits in the utilization of the differently processed cowpeas, which need further investigation.

## Introduction

In the advent of climate change, in the arid and semi-arid tropical farming systems, ecologically adaptable native legumes could provide cost-effective, sustainable solutions to increasingly restricted and erratic local commercial plant protein feed supply chains (Ameen et al., 2005; Adino et al., 2018). There is wide quantitative and qualitative variation among legume species and their cultivars in protein content (Gilani et al., 2005, 2011), and in antinutritional secondary compounds (Garry et al., 2007; Jezierny et al., 2011). Typical of the legumes, cowpea protein is rich in lysine and tryptophan, though relative to animal protein, it is deficient in the sulphur containing amino acids (methionine, cysteine) (Khattab and Arntfield, 2009; Frota et al., 2017). Structural details relevant to the utilization of the main cowpea storage protein, vicilin, were previously described by Kimura et al. (2008), Oliveira et al. (2011), and Rocha et al. (2014). Through impaired digestion (Garry et al., 2007), and by endogenous wastage via damaged gut epithelial cells and secretory protective mucoprotein (Brenes et al., 2004), antinutritional factors (ANFs) may aggravate the inferior protein quality of native legume-based diets (Sauvant et al., 2004; National Research Council, 2012). Therefore, for maximal dietary efficacy, native legumes require tailored thermal (Farran et al., 2001) or biological (Malomo et al., 2013) processing, optimally calibrated for effective deactivation of ANFs, without deleterious impact on protein quality.

Nutrigenetic studies suggest the pig genotype may be important in its capacity to digest and metabolize nutrients from typically chemically complex novel diets. Previous studies suggested different gut morphology (Barea et al., 2011) and profiles of gut microbiota (Fairbrother et al., 2005; van der Meulen et al., 2010; Rist et al., 2013). The genotype also determines the nutrient requirement (Fontanesi et al., 2015). Such genetic differentiation in nutritional traits should be expected after prolonged natural (native pigs) or artificial (exotic pigs) selection in different production systems.

The objective of the present study was to examine the effects on nutrient digestibility and N utilization of processing cowpeas by either sprouting or roasting, for feeding as the primary plant protein source in maize-based diets for Windsnyer (W), Large White × Landrace (LW), and Windsnyer × Large White × Landrace (W × LW × LR) crossbred growing pigs.

## Methods and materials

### Cowpea processing and preparation of experimental diets

Table 1 shows the chemical composition and trypsin inhibitor activity of raw, sprouted, and roasted cowpeas. Cowpeas were bulk-germinated by 12-h pre-soaking in water and 4-day open-air sprouting at ambient conditions, and rapid sun drying. Cowpeas were roasted by placing 20 kg lots of in a cylindrical (L = 1.5 m; Diameter = 0.50 m) manually rotating, cast-iron, gas-heated drum. The procedure involved pre-heating the drum to an initial constant maximal empty interior temperature of 150 °C, followed by introduction of cowpeas for 20-min roasting, to a grain temperature of 105 °C.

**Table 1:** Chemical composition and trypsin inhibitor activity of raw, sprouted, and roasted cowpeas

Components (dry matter basis)		Cowpeas	
	Raw	Sprouted^a^	Roasted^b^
Trypsin inhibitor activity (inhibitor units mg ^−1^)	5.6	4.6	2.3
Dry matter (g kg ^−1^)	934	933	934
Organic matter (g kg ^−1^)	879	886	893
Ash (g kg ^−^1)	55	47	41
Crude Protein (g kg ^−1^)	258	291	260
Ether Extract (g kg ^−1^)	157	92	131
ADF (g kg ^−1^)	125	187	134
NDF (g kg ^−1^)	373	367	240
Amino acids (g/100 g)			
Tryptophan	0.29	0.23	0.30
Arginine	1.38	1.97	1.84
Serine	0.80	1.27	1.19
Aspartic acid	1.34	2.67	2.59
Glutamic acid	2.48	3.77	3.81
Glycine	0.65	0.93	0.91
Threonine	0.53	0.87	0.86
Alanine	0.65	1.13	0.97
Tyrosine	0.27	0.61	0.9
Proline	0.69	1.13	0.95
HO-Proline	0.04	0.08	0.06
Methionine	0.22	0.32	0.32
Valine	0.80	1.18	1.12
Phenylalanine	0.94	1.37	1.3
Isoleucine	0.73	1.03	1.03
Leucine	1.25	1.82	1.76
Histidine	0.62	1.11	1.32
Lysine	1.38	1.68	1.93

^a^Sprouting; 12-h pre-soaking, 4-day open-air sprouting at ambient conditions

^b^Roasting: cylindrical (L = 1.5 m; Diameter = 0.50 m) manually rotating, cast-iron, gas-heated drum, 20 kg cowpeas, initial maximal constant interior drum temperature 150 °C, 20-min roasting to internal drum temperatures at sampling 105 °C

^*^Trypsin inhibitor activity (TIA) was determined according to the American Oil Chemists’ Society official (AOCS) (1998; method Ba 12–75)

Maize grain, the RCP, RSCP, and SCP lots which were used in constituting the dietary treatments were hammer-milled (Jacobson hammer mill, model P160 Teardrop 10HP, China) through a 3-mm screen and mixed (MORHLANG VERTA MIX, 1200VM, USA) for 20 min in 1-tonne lots including amino acid, mineral, and vitamin supplements. The iso-nutrient growing balanced diets are presented in Table 2.

**Table 2:** Ingredient, nutrient, and energy composition of raw, sprouted, and roasted and cowpea diets

Composition		Diets	
	Raw	^a^Sprouted	^b^Roasted
*Ingredients (% as fed)*			
Maize meal	56.8	63.6	57.1
Cowpea meal	40.4	33.6	40.1
*Pig grower micronutrient pack	2.8	2.8	2.8
Total	100.0	100.0	100.0
*Analised chemical and calculated energy composition (DM basis)*			
Dry matter (g kg ^−1^)	932	933	934
Organic matter (g kg ^−1^)	851	886	847
Ash (g kg ^−1^)	8.1	4.7	4.7
Acid detergent fibre (g kg ^−1^)	59	65	63
Neutral detergent fibre (g kg ^−1^)	165	197	252
Fat (ether extract) (g kg ^−1^)	30	29	30
^c^Metabolizable Energy (MJ kg^1^)	17.3	17.3	17.3
Crude protein (g kg ^−1^)	150	150	150
Lysine (g/100 g DM)	1.0	0.8	1.0
Methionine (g/100 g DM)	0.3	0.3	0.2
Calcium (g kg ^−1^)	5.4	5.4	5.2
Phosphorus (g kg ^−1^)	3.3	3.4	3.4

^a^Sprouting; 12-h pre-soaking in water in water, 4-day open-air sprouting at ambient conditions

^b^Roasting: cylindrical (L = 1.5 m; Diameter = 0.50 m) manually rotating, cast-iron, gas-heated drum, 20 kg cowpeas, initial maximal constant interior drum temperature 150 °C, 20-min roasting to 105 °C terminal grain temperature

^c^Metabolizable energy (MJ kg ^−1^) = 1000 × DE – 0.68 × CP (Noblet and Perez, 1993, Eq. 43), where digestible energy (MJ kg ^−1^) = 4.168—9.1 × Ash + 1.9 × CP + 3.9 × EE—3.6 × NDF (Noblet and Perez, 1993, Eq. 23)

^*^Trouw Nutrition, South Africa, containing nutrients per kg of supplement: vitamin A-6500 IU, vitamin D3-1200 IU, vitamin E equivalent-400U, vitamin K3 (43%)-0.0002 g, vitamin B1 (Thiamine Mononitra)-0.00015 g, vitamin B2 80% (Riboflavin)-0.00045 g, niacin (99.5%)-0.0025 g, calcium pantothenate (98%)-0.0012 g, vitamin B12 (1 g/kg)-0.0000003 g, vitamin B6 (98% pyridoxine HCL)-0.00025 g, choline (Chloride 60%)-0.019048 g, folic acid (96%)-0.00006 g, biotin (2%)-0.000005 g, L-lysine 98%-0.00019 g, DL-methionine (98%)-0.02 g, phytase (10 000 FTU/g)-0.005 g, manganese (Manganese Sulphate 31%)-0.004 g, zinc (Zinc So4-Mono 35.5%)- 0.001 g, copper (Copper So4-25.2% Penta)-0.0125 g, iodide (Potassium Iodide 76.45)-0.0001 g, ferrous (ferrous S0_4_-30% Mono)-0.01 g, selenium (Sodium Selenite 4.5%)-0.00003 g, limestone powder-0.1782 g, mono dicalcium phos. (21%)-0.8 g, salt-0.6 g

### Determination of dietary nutrient digestibility and N balance

The digestibility of dietary components was determined using both in vitro (*dry matter*) and in vivo (*fibre*, *nutrients*) techniques. In vitro dry matter digestibility (IVDMD) evaluation was included to separately predict the effects of cowpea processing on compartmental, upper versus lower gut digestion, while the in vivo evaluation enabled the evaluation of dietary effects on intake, on the apparent total tract digestibility of the complete diet and specific chemical components, and effects on measured and calculated parameters of N utilization.

### In vitro dry matter digestibility

The stepwise in vitro pig digestion procedure of Boisen and Fernández (1997) was adapted for micro (5 g) sample digestion in a completely randomized design with 14 replications per sample. Samples were milled through a 1-mm sieve, after which they were oven-dried to a constant weight in a 105 °C, forced-air oven. Samples were then dry-cooled in a desiccator. Approximately 0.5 g samples were weighed into similarly dried Ankom® F57 filter bags which had been pre-rinsed in pure acetone (Acetone for HPLC, ≥ 99.8% (Sigma-Aldrich® product) 34,850). Empty and sample filter bags were pre-weighed and suspended in digestive media within 250-ml glass digestion bottles immersed in a shaking water bath (CNW Model, WBS 450-B) set at 39 °C. Samples were digested in a setup which accommodated 7 treatments × 250-ml digestion bottles in a run, each bottle holding (7 sample + 1 blank (no sample)) filter bags. Given the small micro substrate samples and the relatively large filter bag surface, empty bags were considered necessary to correct for potentially treatment-dependent exchange of fine, non-digestible particulate matter, including particle attachment to the filter bag matrix, to effectively account for the net flux of the fine particles. The digestion procedures were follows: Step 1 (gastric digestion): 87.5 ml phosphate buffer (pH 7.2, 0.1 M, pH 6.0), 35 ml 0.2 M HCl, pH adjusted to 2.0 using 1 M HCl/M NaOH solutions, 3.5 ml aliquot freshly prepared pepsin solution [10 mg/ml pepsin (Pepsin from porcine gastric mucosa powder, ≥ 250 units/mg solid, (Sigma148 Aldrich® product P7000)], 1.7 ml of a chloramphenicol solution (0.5 g Chloramphenicol ≥ 98% (HPLC) (Sigma-Aldrich® product C0378, per 100 ml ethanol), 2-h digestion in a 39 °C shaking water batch. Step 2 (small intestine digestion); after the pepsin digestion, pH adjusted to 6.8 by adding 35 ml of sodium phosphate buffer solution (0.2 M, pH 6.8), 17.5 ml NaOH (0.6 M, pH 13.8), 3.5 ml aliquot freshly prepared pancreatin solution containing 50 mg pancreatin [(Pancreatin from porcine pancreas) (Sigma-Aldrich® product P3292)], 5-h digestion in a 39 °C shaking water batch. Filter bags sequentially gently rinsed in warm tap water, 95% ethanol, and 99% acetone and forced-air oven-drying to constant weight at 105 °C over 24 h for calculation of the in vitro dry matter digestibility (IVDMD). Step 3 (colon or large intestine digestion): step 2 media discarded, 218.75 ml freshly prepared phosphate buffer (0.1 M, pH 4.8), 1.75 ml Viscozyme [(Viscozyme® L, mixture of beta-glucanase, pectinase, hemicellulase, and xylanase enzymes) (Sigma-Aldrich® product V2010)], 18-h digestion in a 39 °C shaking water batch. Filter bags were gently sequentially similarly rinsed and dried for calculation of the IVDMD.

### In vivo digestibility

Nine male pigs, three each of Windsnyer (W), Large White (LW) × Landrace (LR), and 3-way crossbred (W × LW × LR) pig genotypes, were used. The pigs were bred at the Agricultural Research Council-Irene Pig Breeding Unit, from where they were selected from different litters born after natural sow mating, which had been weaned onto a commercial weaner diet at 4 weeks. Mature weights for the parent commercial (LW and LR) and the indigenous, W-type pure breed populations were previously estimated between 300 and 350 kg and 100 and 150 kg, respectively (Kanengoni et al., 2015) . At the start of the experiment, the W, LW × LR, and W × L W × LR groups weighed in at 11 ± 1.15 kg, 14 ± 1.15 kg, and 12 ± 1.15 kg live weight, respectively. The terminal weights were 27.3 ± 1.15 kg, 28.3 ± 1.15 kg, and 27.01 ± 2.0 kg respectively. Prior to the trial, all pigs received a 1-ml subcutaneous injection of Ivomec antiparasitic drug (Reg. No. G2858). The trial was set up in a naturally ventilated house in which each pig was placed within a 57 cm × 118 cm metabolism cage fitted with individual feeders and nipple drinkers. Pigs of the three genotypes were randomly assigned to test diets in a 3 (diet) × 3 (genotype) factorial experiment within three Latin squares, each square with one pig of each genotype rotated through the diets in three feeding periods, in a balanced crossover experiment. The first 7 days of each feeding period in a Latin square were used to adapt the pigs to the dietary treatments, followed by 5 days of measuring the voluntary feed intake, and total faeces and urine collection. Faeces and urine collection and sampling were performed between 08:00 and 09:00 h. The daily urine was collected in 50 ml of 20% HCl, to prevent N volatilization AOAC. 2005. Faecal and urine samples were stored at − 04 °C until analyzed. Frozen faecal samples were dried in a forced-air oven to constant weight at 60 °C, and ground to pass through a 3-mm screen AOAC. 2000.

Feed intake, nutrient digestibility, and nitrogen balance parameters were estimated from the difference between dietary intake and excretion in faeces and urine, and the intake and N parameters expressed on a metabolic body weight (LW^0.75^) basis.

### Chemical analyses

Feed samples were hammer-milled through a 1-mm sieve. Dry matter was determined using the AOAC (2000; method 976.050). Ash was determined using the AOAC (2000; method 923.03). Nitrogen was determined using the micro-Kjeldahl method (AOAC, 2000; method 976.05). Ether extract (EE) was determined by soxlet extraction (AOAC, 2000; method 920.39). Neutral detergent fiber (NDF) and acid detergent fiber (ADF) were analyzed according to Goering and Van Soest (1970).

### Mathematical and statistical analysis

The nitrogen balance parameters are calculated as outlined in Table 3.

**Table 3:** Definitions and equations for nitrogen balance parameters

Component	^a^Formula for estimation
Nitrogen intake (NI)	(N feed/100) × daily feed intake
Faecal nitrogen output (FNO)	(N faeces/100) × daily faecal output
Urinary nitrogen output (UNO)	(N urine/100) × daily urine output
Total nitrogen excretion (TNE)	FNO + UNO
Nitrogen retention (NR)	NI – TNE
Absorbed nitrogen (AN)	NI – FNO
Apparent nitrogen digestibility (ND)	AN/NI
Nitrogen utilization (NU)	NR/NI × 100
The biological value of feed protein (BVFP)	NR/ND × 100

^a^Otto et al. (2003) and Patráš et al. (2009)

The IVDMD digestibility coefficients were subjected to one-way ANOVA in using the GLM of MINITAB software (Version 17.0), based on the model:

$${Y}_{ij} = \mu + {T}_{i}+ {\varepsilon }_{ij}$$

where:

*Y*_*ij*_: = observation.
*µ*: = overall mean.
*T*_*i*_: = effect of the *i*^th^ processing.
*Ɛ*_*ij*_: = random error.

The in vivo measurements of dry matter, fibre, and nutrients digestibility coefficients and of N utilization parameters were subjected to ANOVA in a factorial model which included the fixed effects of the pig genotype, diet, Latin square, periods within Latin squares, the random effects of the animals within Latin squares, and the pig genotype × cowpea diet interaction;

$${Y}_{ijklmn} = \mu + {G}_{i}+{D}_{j}+ {S}_{k}+ {P}_{l}+{A}_{m}+ {(G x D)}_{ij} + {\varepsilon }_{ijklmn}$$

where:

*Y*_*ijklmn*_: - the *n*^th^ observation
*µ*: - the overall mean
*G*_*i*_: - effect of the *i*^th^ pig genotype (*i* =1,2,3)
*D*_*j*_: - effect of the *j*^th^ diet (*j*= 1,2,3)
*S*_*k*_: - effect of the *k*^th^ Latin square (*k* = 1,2,3)
*P*_*i*_: - effect of the *l*^th^ period within Latin squares (*l*= 1,2,3)
A_m_: - effect of the *m*^th^ animal within Latin squares (*m* = 1,2,3)
*(G x D)*_*ij*_: the pig genotype × diet interaction
*Ɛijlmnk*: - the residual error

Tukey’s test was used to compare means where significant (*P* ≤ 0.05) treatment effects occurred.

## Results

### In vitro digestibility

Table 4 shows effects of cowpea processing on in vitro dietary DM digestibility (IVDMD). The roasting cowpeas reduced the dietary step-3 IVDMD compared to sprouting, with no (*P* > 0.05) processing effect on step-2, and the total IVDMD.

**Table 4:** In vitro digestibility of raw, roasted and sprouted growing pig diets

	N	^b^Cowpea diets				
		Raw	Sprouted	Roasted	SEM	Significance
^a^In vitro dry matter digestibility						
Steps 1–2	14	0.50	0.52	0.48	0.00486	ns
Step 3	14	0.38^ab^	0.35^b^	0.39^a^	0.00605	***
Total	14	0.88	0.86	0.87	0.00336	ns

^a^Step 1 (gastric digestion): 87.5 ml phosphate buffer (pH 7.2, 0.1 M, pH 6.0), 35 ml 0.2 M HCl, pH adjusted to 2.0 using 1 M HCl/M NaOH solutions, 3.5 ml pepsin solution (10 mg/ml pepsin (Pepsin from porcine gastric mucosa powder, ≥ 250 units/mg solid (Sigma-Aldrich® product P7000), 1.7 ml of a chloramphenicol solution (0.5 g Chloramphenicol ≥ 98% (HPLC) (Sigma-Aldrich® product C0378, per 100 ml ethanol), 2 h digestion. Step 2 (small intestine digestion); after the pepsin digestion, pH adjusted to 6.8 by adding 35 ml of sodium phosphate buffer solution (0.2 M, pH 6.8), 17.5 ml NaOH (0.6 M, pH 13.8), 3.5 ml aliquot freshly prepared pancreatin solution containing 50 mg pancreatin (Pancreatin from porcine pancreas (Sigma-Aldrich® product P3292), 5-h digestion. Step 3 (colon or large intestine digestion): step 2 media discarded, 218.75 ml freshly prepared phosphate buffer (0.1 M, pH 4.8), 1.75 ml Viscozyme (Viscozyme® L, mixture of beta-glucanase, pectinase, hemicellulose and xylanase enzymes (Sigma-Aldrich® product V2010), 18 h digestion

^b^12-h soaking, 1-, 2-, 3-, and 4-day open-air sprouting, sun-dried

^ab^Within a row with different letter superscripts are significantly different (*P* < 0.05)

*SEM* standard error of the mean

^*^Significant at *P* < 0.05; *P* > 0.05—not significant (ns)

### In vivo evaluation

Table 5 shows treatment effects on in vivo diet utilization. On metabolic weight basis, feed consumption was higher on the RCP compared to the roasted diet (*P* < 0.05). Sprouting and roasting significantly reduced the apparent dry matter and ash digestibility. Ash and ADF digestibility were lower for LW × LR compared to W × LW × LR pigs.

**Table 5:** Dietary intake and nutrient digestibility of raw, roasted, and sprouted cowpea-maize diets by different pig genotypes

Treatments		Intake			Digestibility					
		g/day	g/kg LW	g/kg LW^0.75^	DM	CP	Ash	Fat	ADF	NDF
^3^Genotype	Cowpea diet									
LW × LR	Raw	1169.8	44.9	101.5	0.9	0.91	0.16	0.071	0.36	0.66
	Roasted	1067.2	65.5	131.5	0.85	0.89	0.2	0.102	0.41	0.71
	Sprouted	1034.8	49.3	105.4	0.83	0.88	0.17	0.077	0.42	0.71
W	Raw	1025.8	75.3	144.6	0.88	0.88	0.25	0.095	0.29	0.51
	Roasted	944.5	47.1	99.7	0.81	0.89	0.17	0.065	0.34	0.74
	Sprouted	1019.4	40.2	90.2	0.84	0.9	0.13	0.071	0.4	0.71
W × LW × LR	Raw	1149.9	57.8	122.2	0.89	0.91	0.22	0.087	0.27	0.46
	Roasted	1058..5	42.8	95.4	0.86	0.89	0.16	0.078	0.27	0.49
	Sprouted	1075.5	71.7	141.1	0.82	0.9	0.23	0.094	0.28	0.48
SEM		17.00	1.19	2.13	0.006	0.000238	0.367	0.214	0.795	2.06
Diet (D)										
Raw		1115.2	59.3^a^	122.6^a^	0.89^a^	0.9	0.21^a^	0.084	0.36	0.61
Roasted		1023.4	51.7^b^	108.8^b^	0.84^b^	0.89	0.18^b^	0.082	0.33	0.63
Sprouted		1043.2	53.7^ab^	112.2^ab^	0.83^b^	0.89	0.18^b^	0.081	0.33	0.57
SEM		24.1?	1.69	3.01	0.243	0.003	0.519	0.303	1.12	2.91
Genotype										
W		996.6	54.2	111.4	0.86	0.89	0.19^ab^	0.081	0.34^ab^	0.65
LW × LR		1090.6	53.2	112.8	0.85	0.89	0.18^b^	0.084	0.30^b^	0.55
W × LW × LR		1094.6	57.4	119.5	0.86	0.9	0.20^a^	0.086	0.37^a^	0.62
SEM		24.1	1.69	3.01	0.243	0.003	0.519	0.303	1.12	2.91
Period										
1		1056.2	70.8^a^	139.1^a^	0.85	0.89	0.23^a^	0.098^a^	0.40^a^	0.70^a^
2		1043.1	51.4^b^	109.0^b^	0.85	0.89	0.19^b^	0.076^b^	0.34^b^	0.64^a^
3		1082.6	42.7^c^	95.7^c^	0.85	0.90	0.15^c^	0.073^b^	0.27^c^	0.48^b^
SEM		24.1	1.69	3.01	0.243	0.00336	0.519	0.303	1.12	2.91
*P* values										
Diet		0.093	0.046	0.039	0.003	0.327	0.002	0.771	0.165	0.565
Genotype		0.068	0.344	0.281	0.593	0.174	0.036	0.186	0.014	0.137
Latin square		0.093	0.046	0.039	0.651	0.87	0.002	0.771	0.165	0.565
Period		0.635	0.000	0.000	0.000	0.000	0.000	0.000	0.000	0.001
Animal		0.048	0.344	0.281	0.174	0.184	0.036	0.186	0.014	0.137
Diet × genotype		0.231	0.068	0.067	0.413	0.332	0.435	0.253	0.543	0.426

^1^Sprouting; 12-h pre-soaking in water in water, 4-day open-air sprouting at ambient conditions

^2^Roasting: cylindrical (L = 1.5 m; Diameter = 0.50 m) manually rotating, cast-iron, gas-heated drum, 20 kg cowpeas, initial maximal constant interior drum temperature 150 °C, 20-min roasting to 105 °C terminal grain temperature

^abcd^Within factors or their interaction, treatment means with different letter superscripts are significantly different at (*P* < 0.05)

^*3*^*W* Windsnyer, *LW* Large White; *LR* Landrace

*SEM* standard error of the mean

^*^Significant at *P* < 0.05; *P* > 0.05—not significant

Treatment effects on nitrogen balance parameters scaled to the pig metabolic weight are shown in Table 6. Genotype × diet interactions were significant for nitrogen intake (*P* < 0.0001), digestible N (*P* = 043), urinary nitrogen output (*P* < 0.0001), faecal nitrogen output (*P* < 0.0001), total nitrogen excretion (*P* < 0.0001), and nitrogen retention (*P* < 0.001). The biological value of feed protein was higher (*P* < 0.05) for W pigs, compared to W × LW × LR pigs.

**Table 6:** Nitrogen balance from raw, roasted, and sprouted cowpea-maize diets by different pig genotypes

Treatment		N balance parameters								
		NI	^4^ND	UNO	FNO	TNE	AN	NR	NU	BVFP
		g/kg W^0.75^	g/kg W^0.75^	/kg W^0.75^	g/kg W^0.75^	g/kg W^0.75^	g/kg W^0.75^	g/kg W^0.75^	%	%
^3^Genotype(G)	Cowpea diet (D)									
LW × LR	Raw	2.1^d^	0.89^ab^	0.11^ cd^	0.18^c^	0.29^b^	1.9^ cd^	7.4^ cd^	34.6	39.2
	Roasted	3.1^a^	0.88b	0.19^ab^	0.32^a^	0.52^a^	2.6^ab^	7.6^ cd^	32.7	46.2
	Sprouted	2.5^bc^	0.90^ab^	0.08^d^	0.28^ab^	0.37^b^	2.1^abcd^	8.6^bcd^	34.9	39.4
W	Raw	3.0^ab^	0.88^ab^	0.22^a^	0.34^a^	0.56^a^	2.5^abc^	11.4^a^	37.6	41.1
	Roasted	2.3^ cd^	0.89^ab^	0.15^bc^	0.25^bc^	0.40^b^	1.9^ cd^	8.7^bcd^	36.9	44.6
	Sprouted	2.1^d^	0.91^a^	0.17^abc^	0.20^bc^	0.37^b^	1.7^d^	7.2^d^	34.2	40.8
W × LW × LR	Raw	2.6a^bc^	0.91^a^	0.15^bc^	0.22^bc^	0.38^b^	2.2^abcd^	9.0^bc^	34.8	40.2
	Roasted	2.2^d^	0.90^ab^	0.13^bc^	0.23^bc^	0.36^b^	1.9^ cd^	7.5^ cd^	33.1	41.6
	Sprouted	3.3^a^	0.89^ab^	0.22^a^	0.32^a^	0.55^a^	2.8^a^	10.9^a^	32.9	39.5
SEM		0.477	0.000238	0.00470	0.00544	0.00719	0.0441	0.115	0.510	6.86
Diet										
Raw		2.6	0.90	0.17	0.25	0.41	2.2	9.3	35.6	42.4
Roasted		2.6	0.89	0.16	0.27	0.43	2.1	8.9	34.3	41.1
Sprouted		2.7	0.89	0.16	0.27	0.43	2.2	8.9	34.1	40.7
SEM		0.07	0.003	0.007	0.008	0.010	0.06	0.16	7.210	9.70
Genotype										
W		2.5	0.89	0.18^a^	0.27	0.45^a^	2.1	9.1	36.3	44.2^a^
LW × LR		2.6	0.89	0.13^b^	0.26	0.39^b^	2.2	8.8	34.1	40.1^ab^
W × LW × LR		2.7	0.90	0.17^a^	0.26	0.43^ab^	2.3	9.2	33.6	39.9^b^
SEM		0.07	0.003	0.007	0.008	0.0102	0.062	0.16	7.21	9.70
Period										
1		3.1^a^	0.89	0.21^a^	0.33^a^	0.54^a^	2.6^a^	10.9^a^	34.4	41.6
2		2.4^b^	0.89	0.13^b^	0.25^b^	0.38^b^	2.1^b^	8.8^b^	35.5	42.1
3		2.1^c^	0.90	0.14^b^	0.20^c^	0.34^b^	1.8^b^	7.4^c^	34.0	40.4
SEM		0.07	0.003	0.007	0.008	0.010	0.062	0.16	7.21	9.70
*P* values										
D		0.870	0.327	0.964	0.255	0.540	0.883	0.285	0.382	0.590
G		0.184	0.298	0.001	0.968	0.023	0.118	0.376	0.101	0.032
Latin square		0.870	0.327	0.964	0.255	0.546	0.883	0.285	0.382	0.577
Period		0.000	0.174	0.000	0.000	0.000	0.000	0.000	0.460	0.590
Animal		0.184	0.298	0.001	0.968	0.023	0.118	0.376	0.101	0.032
D × G		0.000	0.043	0.000	0.000	0.000	0.000	0.000	0.609	0.729

^1^Sprouting; 12-h pre-soaking in water in water, 4-day open-air sprouting at ambient conditions

^2^Roasting: cylindrical (L = 1.5 m; Diameter = 0.50 m) manually rotating, cast-iron, gas-heated drum, 20 kg cowpeas, initial maximal constant interior drum temperature 150 °C, 20-min roasting to 105 °C terminal grain temperature

^abcd^Within factors or their interaction, treatment means with different letter superscripts are significantly different at (*P* < 0.05)

^*3*^*W* Windsnyer, *LW* Large White; *LR* Landrace

^4^Nitrogen intake (NI)—(N feed/100) × daily feed intake; faecal nitrogen output (FNO)—(N faeces / 100) × daily faecal output; urinary nitrogen output (UNO)—(N urine / 100) × daily urine output; total nitrogen excretion (TNE) FNO + UNO—nitrogen retention (NR)—NI – TNE; absorbed nitrogen (AN)—NI – FNO; apparent nitrogen digestibility (ND)—(AN / NI); nitrogen utilization (NU)—NR / NI × 100; the biological value of feed protein (BVFP)—NR / ND × 100

*SEM* Standard error of the mean

^*^Significant at *P* < 0.05; *P* > 0.05—not significant

## Discussion

In the present study, a combination of compartmental pig gut in vitro (dry matter digestibility) and apparent in vivo (fibre, nutrient digestibility, N balance) evaluation was employed to estimate the effects of sprouting versus roasting of cowpeas on diet utilization by weaned pigs of diverse genotypes*.* Low partial step-3 IVDMD of sprouted compared to roasted cowpeas reflected the cumulative effect of greater quantitative steps 1–2 digestion, and of escape residues, mainly consisting of poorly or slowly digestible, fibrous cell-wall structural matter in the sprouts. Unfortunately, the micro-gravimetric digestion setup excluded molecular insight into the nature of the biochemical effects, which could have important implications on pig nutrition. In contrast, both roasting and sprouting cowpeas similarly reduced the in vivo apparent ash and DM digestibility, which, particularly for DM, implied outflow rate-dependent escape of slowly digested plant embryonic secondary tissues in cowpea sprouts, and of cross-linked compounds in roasted cowpeas from all including spontaneous disintegration, and the endogenous enzyme hydrolytic (steps 1–2) or microbial (step 3) fermentative digestion. In pigs, the fate of feed passage and nutrient digestibility in the gut is subject to the dietary chemical and physical properties, such as the type and levels of dietary fibre, and the feeding level (Le Goff et al., 2002). The DM for the RCP, SPC, or RSCP diets that escaped the foregut could be of variable bulkiness, which is known to influence gut emptying in growing pigs (Kyriazakis and Emmans, 1995). However, in vivo, less escape, particularly of fibrous matter, should be expected, since, compared to a commercial product such as Viscozyme, the greater diversity of pig gut microbial fibrolytic activity (Fushai et al., 2019) should confer higher digestibility of fibrous compounds in the colon. Fibrolytic advantage of the indigenous pig gut was previously reported from genomic evaluation of faecal microbiota (Kanengoni et al., 2015). We hypothesized that superior fibrolytic capacity may also theoretically present a mechanism for broader tolerance of toxic feed ANFs. In the present study, W × LW × LR had higher ADF digestibility than the LW × LR pigs, though both had similar ADF digestibility to the W pigs. A similar, though quantitative, pattern was observed for NDF.

Metabolic size-scaled feed consumption was higher on the RCP compared to the SPC and RSCP diet (*P* < 0.05). We interpreted high intake to be due to energy deficit–induced upregulation of intake to compensate for inefficient digestion and, or metabolism of nutrients.

In livestock production, efficient utilization of protein depends on understanding the optimum levels that meet protein needs for growth, reproduction, and maintenance (Paul et al., 2007). Imprecise supply of protein cannot support optimal protein deposition, and is detrimental to pig performance (Whittemore et al., 2001; Noblet et al., 2001; Nørgaard et al., 2014). Excess dietary protein increases waste nitrogen excretion through the urine (Carpenter et al., 2004). The conservation of dietary N is therefore critical to meet both environmental and economic objectives (Rotz, 2004). To achieve these objectives, diet formulation should strike the correct balance between the quantitative and qualitative protein intake and the animal requirement, to minimize N excretion, and optimize animal productivity. Aquilani et al. (2019) highlighted that slow growing pigs have lower protein requirements compared to that of lean genotypes, and should theortically be more tolerant to inferior dietary protein.

On standard, multiphase-feeding diets, like poultry, commercial growing pigs are highly N efficient, as high as 40% nitrogen use efficiency (Rotz, 2004). In the current study, NU ranged between 34.0 and 35.5%, with BVFP (%) of 39. 2–46.2%. The values at the low end of these parameters are for inferior protein quality in terms of digestion and/or imbalance in the profile absorbed amino acids, and reflect poor tissue utilization (Smiricky et al., 2002). Significant dietary N wastage occurs through both protein indigestion and poor tissue protein assimilation (Rotz, 2004). Depending on the diet, protein digestion is highly variable, as is the partition of N excreted via the urinary, relative to the  faecal routes (Ball et al., 2013).

Complex gut-systemic exchange of endogenous and dietary protein, amino acids, and urea occurs, which is dependent on the diet quality (Ball et al., 2013) and intake (Ball et al., 2013). In the current study, ND was overall high for the RCP (90%), sprouted (89%), and roasted (89%) cowpea diets. In vitro, El-Jasser (2011) reported 75–79% protein digestibility of sprouted cowpeas. In vivo, sprouting (Urbano et al., 2005) and thermal processing (Doblado et al., (2007) of cowpeas improved protein digestibility, though excessive heating reduced its digestibility, reflecting negative effects of non-enzymatic (Maillard) reactions between the reducing sugars and proteins, and thermally induced amino acid cross-linking (Tuśnio et al., 2017). Unfortunately, in the present study, neither in vitro nor in vivo evaluation measured the ileal protein or amino acid digestibility, to properly predict processing effects on protein quality (Mosenthin et al., 2000; Święch, 2017).

Legume seeds contain ANFs that affect feed intake and nutrient digestibility and also compromise functions of the liver, kidneys, and intestines (Ndou et al., 2015). Variable dietary DM, protein, and amino acid digestibility should therefore be expected of diets in which the legume feed contains significant antinutritional factors (Kumar et al., 2006; Kayembe, 2013). In the present study, pigs were fed diets equally balanced in N and energy RCP, SPC, or RSCP diets. Chemical analyses suggested significant residual trypsin inhibitors in the sprouted, but less so in the roasted cowpeas.

The pattern of NI across the genotype × diets treatments reflected that of the feed intake. However, different diet × genotype interactions were observed for ND, FNO, TNE, NU, and the NR, which suggested genotype-differentiated N metabolism depending on cowpea processing. Nitrogen extraction (ND) from the gut seemed more efficient in W pigs on the SPC, while the W × LW × LR pigs were similarly efficient in extracting N from the sprouted cowpeas diet, with poor extraction of N in RSCP diet by LW × LR pigs. The pattern of ND either directly reflected the dietary N availability or inversely reflected the endogenous wastage, a function of dietary ANFs. Moreira et al. (2004) reported that the total nitrogen excretion is determined by certain factors, primarily the crude protein content of diet. Canh et al. (1998) reported a 45% reduction in urinary nitrogen excretion when the dietary crude protein content decreased from 16.5 to 12.5%. In the present study, faecal N wastage (FNO) was highest for LW × LR pigs on the RSCP diet, the W × LW × LR on the SPC diet, and the W on the RCP diet and was lowest for the LW × LR pigs on the sprouted cowpeas. Similar to the ND, the pattern of faecal N excretion likely reflected the undigested fraction, along with the endogenous N wastage, both of which are affected by ANFs. Urinary wastage (UNO) was highest for W × LW × LR pigs on the SPC diet and the W pigs on the sprouted cowpeas diet, with the low wastage by LW × LR pigs on the SPC diet. The urine N excretion could reflect excess dietary N or an imbalanced amino acid profile relative to the pig requirement, or a dietary energy deficit which required partial protein utilization for energy. Imbalance in amino acids supplied for protein synthesis for growth and other functions results in catabolism of excess amino acids, with excess N converted to urea, which increase its excretion in urine (Ball et al., 2013). On the other hand, protein indigestion in the upper tract diverts N to colon fermentation (Bindelle et al., 2009). With adequate fermentable energy supply, colon bacteria assimilate both endogenous and dietary N, to lock it and shift excretion from urea in urine to microbial protein in faeces (Bindelle et al., 2009). Hlongwana et al. (2021) confirmed high N excretion through faeces as a consequence of either dietary or endogenous N diverted to microbial protein synthesis, thereby increasing microbial biomass in the hindgut. If energy is deficient, increased colon protein fermentation may also produce toxic nitrogenous metabolites (Tuśnio et al., 2017). The pattern of total excreted N (TNE) was consisted with the summative FNO and UNO, the net effect of which was similarly high total N excretion for LW × LR pigs on the RSCP diet, the W × LW × LR on the sprouted cowpeas diet and the W on the SPC diet compared to other treatment combinations. The AN mirrored the ND, with the highest observed values for W × LW × LR pigs on the SPC diet, and the lowest values observed for W pigs on the SPC diet. The nitrogen which was assimilated into tissues (NR) was highest for W × LW × LR pigs on the SPC diet and for W pigs on the sprouted cowpeas diet, and lowest for W pigs on the SPC diet. Measured by the NU, protein efficiency was not differentiated by the pig genotype, or the diet, and not subject to the genotype × diets interaction. However, measured as the BVFP, protein efficiency was low for W × LW × LR pigs compared to W pigs, which suggested imbalance in either the dietary amino acids, or the protein-energy ratio, in relation to the genotype requirement.

In conclusion, in the present study, the pattern of compartmental and the total IVDMD suggested that, compared to roasting, sprouting increased and/or shifted fibrous substrates and their digestion to the lower gut, without effect on total IVDMD. In contrast, in vivo, roasting and sprouting of cowpeas similarly reduced dietary DM digestibility compared to raw cowpeas, which implied outflow rate-dependent escape from total digestion of slowly digested plant embryonic secondary tissues in cowpea sprouts, and of cross-linked compounds in roasted cowpeas. The W × LW × LR expressed higher capacity to digest ADF than the LW × LR pigs, though both had similar ADF digestibility to the W pigs. Though partly the effect of intake induced differences in outflow rate, the genotype differentiation in capacity to digest different fibre fractions could reflect different microbiota ecosystems in the hindgut. The nitrogen balance responses were characterized by interaction of diet with the pig genotype. Nitrogen extraction from the gut (ND) seemed more efficient in W pigs on the SPC, while that of W × LW × LR pigs was efficient in extracting N from the sprouted cowpeas diet. The LW × LR pigs were inefficient in extracting N from the RSCP diet. The pattern of ND was explained by either different extraction of dietary N or inversely by different endogenous wastage, both functions of dietary ANFs. In the present study, faecal N wastage (FNO) was highest for LW × LR pigs on the RSCP diet, the W × LW × LR on the SPC diet, and the W on the sprouted cowpeas diet and was lowest for the LW × LR pigs on the sprouted cowpeas. The UNO was highest for W × LW × LR and W pigs on the SPC diet, with low wastage by LW × LR pigs on the SPC diet. We attributed this pattern of urine N excretion to excess dietary its imbalance in amino acids relative to the pig requirement, or a dietary energy deficit, which triggered protein utilization for energy. The pattern of TNE was consisted with the summated FNO and UNO, the net effect of which was similarly high total N excretion for LW × LR pigs on the RSCP diet, the W × LW × LR on the SPC diet, and the W on the SPC diet compared to other treatment combinations. The AN mirrored the ND, with the highest observed values for W × LW × LR pigs on the SPC diet, and the lowest values observed for W pigs on the SPC diet. The nitrogen which was assimilated into tissues (NR) was highest for W × LW × LR pigs on the SPC diet and for W pigs on the SPC diet, and lowest for W pigs on the SPC diet. Overall, measured by the NU, protein efficiency was not differentiated by the pig genotype, or the diet, and not subject to genotype × diet interaction. However, measured as the BVFP, protein efficiency was low for W × LW × LR pigs compared to W pigs, which suggested imbalance in either the dietary amino acids or the protein-energy ratio, in relation to the genotype requirement, inefficient nitrogen extraction, or excessive endogenous gut mucous excretion due to antinutrients. The “period” in the experimental setup represented incremental age of the pigs, which increased the NI, UNO, FNO, and NR, with a somewhat inverse trend for the TNE and AN. We concluded that both sprouting and roasting caused chemical changes which decrease the dietary intake and depress mineral, and the DM digestibility. The LW × LR expressed lower digestibility of the more recalcitrant ADF. Pig × genotype interaction with the biological versus thermal cowpea processing resulted in significant variation in pig responses in terms of N balance parameters which relate to different amino acid metabolism, effects which suggested unique digestive and metabolic adaptive traits among the experimental pig genotypes to the diets. The implications need verification in large-scale performance trials, with more extensive dietary chemical characterization.

## Data Availability

There is no additional or supplementary data to the study.
